# Community resource centres to improve the health of women and children in Mumbai slums: study protocol for a cluster randomized controlled trial

**DOI:** 10.1186/1745-6215-14-132

**Published:** 2013-05-08

**Authors:** Neena Shah More, Sushmita Das, Ujwala Bapat, Mahesh Rajguru, Glyn Alcock, Wasundhara Joshi, Shanti Pantvaidya, David Osrin

**Affiliations:** 1 Society for Nutrition, Education and Health Action (SNEHA), Urban Health Centre, Chota Sion Hospital, 60 Feet Road, Shahunagar, Dharavi, Mumbai, 400017 Maharashtra, India; 2 Institute for Global Health, UCL Institute of Child Health, 30 Guilford Street, London WC1N 1EH, UK

**Keywords:** Public health, India, Mumbai, Urban health, Slums, Poverty

## Abstract

**Background:**

The trial addresses the general question of whether community resource centers run by a non-government organization improve the health of women and children in slums. The resource centers will be run by the Society for Nutrition, Education and Health Action, and the trial will evaluate their effects on a series of public health indicators. Each resource center will be located in a vulnerable Mumbai slum area and will serve as a base for salaried community workers, supervised by officers and coordinators, to organize the collection and dissemination of health information, provision of services, home visits to identify and counsel families at risk, referral of individuals and families to appropriate services and support for their access, meetings of community members and providers, and events and campaigns on health issues.

**Methods/design:**

A cluster randomized controlled trial in which 20 urban slum areas with resource centers are compared with 20 control areas. Each cluster will contain approximately 600 households and randomized allocation will be in three blocked phases, of 12, 12 and 16 clusters. Any resident of an intervention cluster will be able to participate in the intervention, but the resource centers will target women and children, particularly women of reproductive age and children under 5.

The outcomes will be assessed through a household census after 2 years of resource center operations. The primary outcomes are unmet need for family planning in women aged 15 to 49 years, proportion of children under 5 years of age not fully immunized for their ages, and proportion of children under 5 years of age with weight for height less than 2 standard deviations below the median for age and sex. Secondary outcomes describe adolescent pregnancies, home deliveries, receipt of conditional cash transfers for institutional delivery, other childhood anthropometric indices, use of public sector health and nutrition services, indices of infant and young child feeding, and consultation for violence against women and children.

**Trial registration:**

ISRCTN Register: ISRCTN56183183

Clinical Trials Registry of India: CTRI/2012/09/003004

## Background

### Rationale

A recent review of developments in urban health over the last 30 years identified four perceptual shifts [1]. First, more than health service inputs are necessary to improve health. Of particular interest are community participation and formation of partnerships with community-based organizations. Second, the emphasis should shift from individuals to communities. Third, there was a growing interest in multi-level determinants of health, including work on poverty, social interactions, the physical environment and services. Fourth, initiatives must go beyond the public sector. Informal settlement communities are heterogeneous [2,3], and health care often involves the private and informal sectors [4-7].

The Society for Nutrition, Education and Health Action (SNEHA), a Mumbai-based non-government organization (NGO), works to improve the health of women and children in disadvantaged communities. We have addressed health care from two directions: on the demand side, by attempting to create informed users of health services who expect higher quality; and on the supply side, by working with public sector health providers (in our city, the Municipal Corporation of Greater Mumbai) to improve the quality of health services [8].

A National Urban Health Mission (NUHM) is due to merge with the existing National Rural Health Mission of the Government of India. It seeks to address the health care needs of the rapidly growing urban population, with a focus on the disadvantaged. A significant change in the proposed strategies is a move from the provider perspective to a more collaborative approach. There is an emphasis on building local capacity and engaging communities in delivery of health care, and on building public-private partnerships to enhance quality of care. Any potentially scalable intervention should fit the National Health Mission agenda: training of link-workers and women’s health committees to carry out community health promotion activities, strengthening linkages between service providers and the community, especially vulnerable groups, regular outreach services to address low access by disadvantaged groups, and public-private partnership.

We have become interested in the potential of community resource centers as nexuses for improving family and community health. There is a tradition of NGOs basing their community work at local resource centers. In some cases the NGOs are small and the resource centers are their headquarters. In others, they are satellite nodes linked with larger central offices: the structural arrangement we aim to test. We estimate that there are about 60 major NGOs working on urban informal settlement development in Mumbai. Prominent groups, including Society for Promotion of Area Resource Centres (http://www.sparcindia.org), Akanksha Foundation (http://www.akanksha.org), Apnalaya (http://www.apnalaya.org), DoorstepSchool (http://www.doorstepschool.org) and Pratham (http://www.pratham.org), have run local community resource centers since the 1980s. These have served purposes as varied as provision of preschool, non-formal and remedial education, vocational training, recreation activities (*khelwadis*), health clinics, care centers for people with disabilities, family counseling, collective savings and loans, and physical space for community interaction. Some organizations, including Apnalaya, Stree Hitkarni, Committed Communities Development Trust (http://www.ccdtrust.org), and Navjeevan (http://www.navjeevan.org), have focused on community health. Their resource centers occupy a range of locations: individual homes, leased spaces, or sites provided by community-based organizations. They are staffed by a combination of volunteers and salaried cadres and are open from 8 to 24 hours daily.

Our previous trial of community mobilization through women’s groups suggested that women were eminently able to articulate their experiences, identify problems and suggest local solutions, but that they hit a wall when they tried to move to community action [9]. To some degree this is a feature of what we call the urban paradox: despite the density of informal settlement populations, contact with people outside one’s immediate area, cultural or kinship group is limited. While women’s groups in rural areas seem to be able to pull together communities for collective action [10,11], groups in urban informal settlements - though probably less poor and more ‘modern’ - often feel that they lack the power to push their agendas with neighbors and health-care providers.

Our idea is that satellite resource centers located in vulnerable areas could be formalized sources of health information and bases for community outreach work. Workers at each center will be members of the SNEHA team, backed by the experience, knowledge, connections and skills of project coordinators and directors: a decentralization in the non-government sector that answers calls for decentralization in the government sector. Information, training, awareness and advocacy events will be cascaded out through the resource centers. The centers will coordinate services such as community-based contraceptive distribution, outreach camps for immunization, counseling services for women facing violence, and day care with supplementary nutrition for malnourished children.

### Aims

Our propositions are that: (i) on the basis of our experience in community mobilization for health, we would like to move to a decentralized community resource center model; (ii) for feasibility given our expertise, we will limit both the intervention and its evaluation to health issues; (iii) although the model is a common one with many potential benefits, we have equipoise on its effect on population health; (iv) we would like to evaluate the model on the basis of outcomes designed to be unambiguous, commonly measured, externally comparable and representative of women’s and children’s health.

## Methods/design

### Setting

The capital of Maharashtra state, Mumbai has a provisional 2011 census population of 12.5 million [12], more than half of whom live in slums. About one-fifth of slum homes have a private toilet, 31% of residents have completed 10 years of education, and the total fertility rate is below the replacement threshold at 1.9 [13]. Public sector care is provided by the Municipal Corporation. Private health care is widely available and ranges from specialty hospitals to informal practitioners. The trial will be conducted in two of the city’s twenty-four municipal wards, each of which has a population of about 700,000, chosen on the basis of poorer human development ranking and a high proportion of slum settlements.

### Trial design

**Figure 1:**
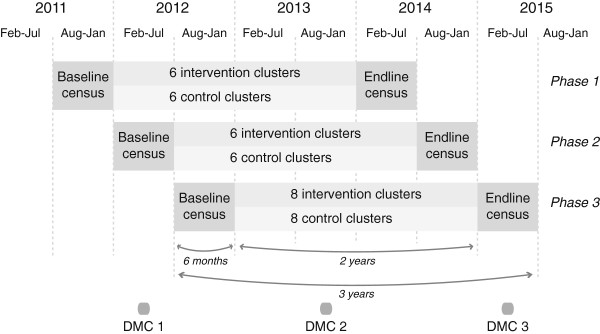
**Trial design.** DMC, data monitoring committee.

The study is a cluster randomized controlled trial in which 20 slum areas will be allocated to have community resource centers and 20 will act as controls. Allocation will be done in three blocks, of 12, 12 and 16 clusters, in a phased design with 6-month intervals between the start of each phase (Figure 1). It has been our experience that, in large cluster randomized controlled trials, instituting interventions simultaneously in all clusters is problematic. The phased design is logistically feasible and, we hope, will allow clear demarcation of intervention start times. We will conduct two rounds of data collection: a baseline census and a census after 2 years of intervention, in which the information is provided mainly by married women aged 15 to 49 years.

### Cluster size and selection

The sample frame will include clusters of approximately 600 households. Where settlements are large, we will divide them into smaller clusters along obvious physical boundaries. One large area may then provide more than one potential trial cluster, but we will try to avoid contiguity to minimize contamination. We think that the likelihood of contamination is limited both because of the urban paradox and because this has been our experience in previous projects. The distribution of vulnerability is not random and we will be able to define a sample frame within particularly deprived city wards. For example, areas in M-East ward are generally more vulnerable than others, with higher proportions of home births and higher mortality rates. The first step will be to identify the informal settlement areas in the chosen wards. This will be done by the data collection team, using their existing knowledge and inputs from the Municipal Corporation, the Tata Institute of Social Sciences, NGOs, and local key informants. The sample for each phase will be based on vulnerability scores derived from a rapid assessment tool [14].

### Participants

Although any resident will be able to participate in the intervention, the resource centers will target women and children, particularly women of reproductive age and children under 5 years of age. Evaluation will be based largely on interviews with married women aged 15 to 49 years.

### Interventions

**Figure 2:**
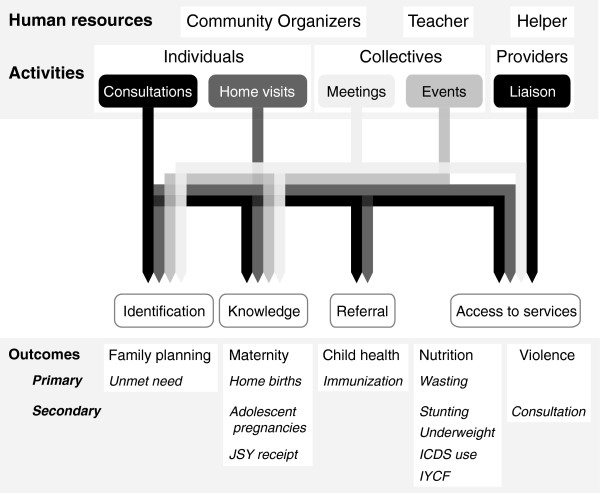
**Pathways to impact for the community resource center model.** ICDS, Integrated Child Development Services; IYCF, Infant and Young Child Feeding

The SNEHA center will be a community space in a rented room within the cluster that it serves. Each center will be a base for three salaried community organizers, a teacher to work on early child development, and a helper to provide services for malnourished children. Each community organizer will cover approximately 200 households, a ratio designed to fit with the proposed coverage of the Urban Social Health Activist proposed for the NUHM. Every two centers will be supervised by a program officer, and every six to eight by a program coordinator. Community organizers will communicate with three constituencies - individual residents, collectives and service providers - through five axes of intervention: consultations, home visits, group meetings, community events and communication with service providers (Figure 2). Within an integrated framework, the priority health issues will be maternity care, family planning, childhood nutrition and health, and violence against women and children.

### Inception

The community organizers will map their working areas and identify key services and institutions. A series of participatory learning and action exercises (micro-planning) will follow, and will be a first step to involving residents in voluntary activities such as community action groups, parents’ and youth groups, and support for the center. Community organizers will lead visits to local NGOs and service providers in order to establish a referral network, avoid duplication of care, and encourage uptake. The findings of the process will be disseminated in the community and a process of participatory planning and monitoring initiated.

### Home visits

Community organizers will maintain a numbered electronic profile of each family in their catchment areas. They will make approximately six home visits daily and record their activities using electronic data capture on smartphones and a database system in CommCare (www.commcarehq.org). They will identify family health needs in each of the four priority areas, provide relevant information, guide and support family members to take action, and reinforce successful experiences through peer learning at group meetings and community events. Their support options will be information and advice, referral and accompaniment to SNEHA or another organization, or direct service provision. Examples of information include sources of maternity care, danger signs, the *Janani Suraksha Yojana* safe maternity incentive, family planning, infant feeding, immunization, and domestic violence. Examples of advice and action include referral to health providers, accompaniment to support access, arrangement of day care for childhood malnutrition, referral for counseling for violence, and provision of contraception through a partnership with the Family Planning Association of India.

### Group meetings

Community organizers will facilitate daily group meetings, at the center or elsewhere in the community. Meetings will follow a participatory action research cycle addressing issues of concern for pregnant women, new mothers and mothers of young children, adolescents, and local stakeholders.

### Community events

With input from SNEHA programs, community organizers will act as local facilitators for rallies, street drama, competitions, and health campaigns. Community members - especially the youth - will be mobilized and trained to participate in community processes and action.

### Service provision

We will work with the Municipal Corporation, the Integrated Child Development Services (ICDS), local NGOs, the police, and community-based organizations to improve availability, access and uptake of services. Community organizers will mobilize community members to increase uptake of existing services such as immunization and medical camps, and will facilitate interaction meetings for community members and service providers. The resource centers will themselves provide some services: nutritional support, early childhood stimulation, medical consultations, and support for women and children facing violence. Because we are concerned about the refractory nature of childhood malnutrition, we are partnering with the ICDS and Child Rights and You (http://www.cry.org) to implement protocols for identification of malnourished children. A teacher and a helper will support children under 5 years of age with moderate or severe acute malnutrition, through supplementary feeding, provision of ICDS take-home rations, immunization and deworming, and growth monitoring, with home follow-up by resource center workers. Concurrently, we will run early child development activities designed in consultation with Mumbai Mobile Creches. Mothers will be sensitized to the health and development needs of their children and involved in center activities through regular home visits and group meetings. Community organizers will be supported by two medical officers with access to basic medicines, and through outreach pediatric camps organized by the Municipal Corporation. Community organizers will identify domestic violence, and SNEHA counselors will be available for support.

## Outcomes

The outcomes will be assessed through a household census after 2 years of resource center operations. The intervention will address women’s and children’s health in general, including the health of adolescents and unmarried women. We have been more selective in terms of evaluation, since outcomes such as family planning, pregnancies, and deliveries are best addressed in the context by focusing on married women. They also include consultations for domestic violence, which are not limited to married women and will be examined through consultation records rather than cross-sectional data.

### Primary outcomes

1. Unmet need for family planning in women aged 15 to 49 years: based on the London Measure of Unplanned Pregnancy, a six-question module that has been tested in urban India [15].

2. Proportion of children under 5 years of age not fully immunized for their ages: based on Indian Academy of Pediatrics recommendations [16].

3. Proportion of children under 5 years of age with weight for height less than 2 standard deviations (SD) below the median for age and sex.

### Secondary outcomes

1. Number of consultations for violence against women or children.

2. Proportion of home deliveries for births in the preceding 1 year.

3. Proportion of pregnancies in the preceding 2 years to women under 19 years under age.

4. Proportion of public sector institutional deliveries for which the *Janani Suraksha Yojana* birth incentive was received.

5. Proportion of children under 5 years of age with height for age less than 2 SD below the median for age and sex.

6. Proportion of children under 5 years of age with weight for age less than 2 SD below the median for age and sex.

7. Proportion of children born in the preceding 2 years who received food supplements, health check-ups, early childhood development intervention, or had their weight measured at ICDS centers.

8. Infant and Young Child Feeding core indicators [17].

a. Early initiation of breastfeeding: proportion of children born in the last 24 months who were put to the breast within 1 hour of birth

a. Exclusive breastfeeding under 6 months: proportion of infants aged <6 months who received only breast milk during the previous day

a. Continued breastfeeding at 1 year: proportion of children aged 12 to 15 months who received breast milk during the previous day

a. Introduction of solid, semi-solid or soft foods: proportion of infants aged 6 to 8 months who received solid, semi-solid or soft foods during the previous day

a. Minimum dietary diversity: proportion of children aged 6 to 23 months who receive foods from four or more food groups

a. Minimum meal frequency: proportion of breastfed and non-breastfed children aged 6 to 23 months who receive solid, semi-solid, or soft foods (but including milk feeds for non-breastfed children) the minimum number of times or more

a. Minimum acceptable diet: proportion of children aged 6 to 23 months who receive a minimum acceptable diet

a. Consumption of iron-rich foods: proportion of children aged 6 to 23 months who receive an iron-rich or iron-fortified food

### Sample size

Based on the phase 1 baseline census in 12 clusters, we estimate that we will achieve interviews with 350 married women aged 15 to 49 years per cluster, and that we will have information on 80 pregnancies in the preceding 2 years, 80 children born in the preceding 2 years, and 120 children over 2 and under 5 years of age. We estimate that we will manage to measure the weights and heights of 150 children under 5 years of age per cluster. The sample size calculations assume two treatment groups, unmatched clusters of approximately equal size, and values of *k* (coefficient of variation of true proportions between clusters) equal in intervention and control groups [18]. Table 1 summarizes expectations for the primary outcomes. Control area proportions and values of *k* are based on the phase 1 baseline census in 12 clusters. The estimates were all made at 80% power. Because the intervention is part of our service delivery program, is of minimal risk, and will be evaluated after 2 years of operations, we are not specifying stopping rules.

**Table 1:** Detectable differences in primary outcomes between allocation groups, with 40 clusters allocated 1:1, intervention:control

**Indicator**	**Estimated records per cluster**	***k***	**Control estimate (%)**	**Intervention estimate (%)**	**Detectable difference (%)**	**Detectable relative difference (%)**
Unmet need for family planning	350	0.11	46	41	−5	11
Incomplete basic immunizations in children under 5 years of age	160	0.22	66	53	−13	17
Weight-for-height >−2SD below median for age and sex in children under 5 years of age	150	0.1	20	16	−4	20

Records per cluster, *k*, and control estimate based on phase 1 data for 12 clusters. *k*, coefficient of variation of true proportions between clusters; SD, standard deviation.

### Randomization

Clusters have been pre-randomized by number (by SD and DO), in blocks of 12, 12 and 16. The randomization plan was created on 25 July 2011 (http://www.randomization.com) using seed 11426, and stored securely. Project staff were not aware of the allocation during the process of consent for cluster inclusion.

### Blinding

Because of the nature of the intervention, allocation is not concealed.

### Data collection

Data are collected in baseline and endline censuses of cluster households. Two teams of six interviewers and one program officer cover a cluster at a time, defining its boundaries, mapping it and numbering the households. Each interviewer is allocated 10 households at a time, preferentially interviewing all married women aged 15 to 49 years. If none lives in a household, or if she is absent at three visits, another adult over 18 years of age is interviewed. The interview enumerates household members, their ages, schooling and livelihoods. It then covers duration of residence, assets and amenities, housing fabric, and faith. Women aged 15 to 49 years provide brief maternity histories and information on family planning. If they have been pregnant in the preceding 2 years, they are asked about antenatal care, delivery location and outcomes, and infant feeding. Information on immunizations and use of the ICDS is collected for all children under 5 years of age. Children are listed and their weights and heights are measured on designated days at the end of each cluster census. Data are entered on smartphones running Open Data Kit (ODK: http://opendatakit.org) on the Google Android operating system (http://www.android.com). A program officer will observe 5% of interviews. The interface includes generation of unique identifiers for women aged 15 to 49 years and children under 5 years of age, automatic skips and validation constraints to minimize error.

### Data management

Data are transferred electronically to a secure ODK Aggregate cloud repository on a password-protected Google Appspot. The dataset is downloaded twice weekly and run through automated error checks. Each week, 50 records (20 to 25% of interviews) are extracted after random numbering, printed on spreadsheets, and re-checked in the field. Data are also checked after download for errors in key fields, and monitoring summaries are produced through do-files written in Stata 12 (StataCorp, College Station, TX, USA; www.stata.com). The definitive dataset contains numerical identifiers for cluster, household number and participant. The names of heads of household and participants are collected during the interviews, but removed after storage. Access to data is restricted to the data manager and analysts. Datasets are backed up weekly on a server and compact discs.

### Interim analysis

A data monitoring committee (DMC) will meet three times (Figure 1) and follow the Data Monitoring Committees: Lessons, Ethics, Statistics guidelines [19]. At the first meeting, in May 2012, the committee considered the protocol, analysis plan, and baseline census from the 12 clusters in phase 1. Key questions were whether the baseline levels of outcome indicators accorded with our projections, and whether allocation was balanced. The second meeting will consider data from all three baseline phases and discuss any need for changes in the intervention approach, and the third will review the analysis plan against interim outcome data.

### Analysis plan

An analysis plan was discussed at the first meeting of the DMC in May 2012, and has been sealed. Presentation will follow the Consolidated Standards for the Reporting of Trials guidelines [20,21], beginning with a trial profile describing numbers of clusters, households, women and children enrolled in the evaluation, a summary of deviations from protocol, and a description of recruitment. Markers of identification will not be retained in analytical datasets. We will present a baseline comparison of allocation groups, summarizing household numbers, socioeconomic descriptors, including the Multidimensional Poverty Index [22], women’s age, education, and duration of residence, and numbers of births, and children under 2 and under 5 years of age. We will compare the primary outcomes between allocation arms, using data from the endline census after 2 years of intervention. Provided that distribution criteria are met, we will use logistic regression with a random effect for cluster [18], adjusted for phase (the second option being generalized estimating equations). We will enter each outcome as a dependent variable and allocation as a binary independent variable. If substantial baseline imbalances between allocation groups in demographic and socioeconomic characteristics are noted at the second DMC, we will include them as independent variables in multivariable models. If there are baseline imbalances in primary and secondary outcomes, we will model change in proportion between baseline and endline.

## Ethical considerations

### Consent

Two levels of consent will be taken: cluster and individual. We will seek signed cluster-level gatekeeper consent for trial inclusion. We will identify cluster gatekeepers using a predefined protocol. The developing ethical consensus on cluster trials suggests that gatekeeper consent may not be mandatory in this case, but we consider gatekeeper consultation important [23]. Involvement in actual program activities will be at individual discretion. Participants will come to know about the resource centers through local presentations, word of mouth, and home visits by community officers. Right to withdraw is implicit in the intervention, since attendance at or involvement with resource center activities requires active participation. Potential respondents for census data collection will be identified at systematic household visits by field investigators. We will provide standardized information about the trial and explain the procedures for anonymizing data. The right to withdraw from an interview will be explained before it begins, and is specified in the consent form. Participants will be recruited on the basis of agreement to be interviewed and signed consent. No monetary compensation will be given to any participant.

### Risk

Although no interview participants will be vulnerable (apart from children under 5 years of age who participate in anthropometry), an ethical issue that may arise is the identification of participants at risk during the household census. We believe that data collectors have a duty of care for participants, in accordance with their abilities. Since the individuals involved in data collection are not health workers, their skills do not extend to management of illness. If it appears to an interviewer that a respondent has a personal or family problem, she will have a duty of care to communicate this and support access to consultation, support which we will facilitate through orientation and provision of information on sources of help. Clear protocols for consultation and support are available to data collectors.

We have identified no specific risks associated with community resource centers themselves. However, the nature of the work will be that women and children at risk will be identified, since this is the point. They may be malnourished, have concerns about family planning or institutional delivery, or be experiencing domestic violence. It is therefore crucial that resource center community workers - and the project as a whole - have clear protocols for addressing such concerns. SNEHA has been working in this domain for over a decade now, and ethical approval has required us to present detailed protocols for training, information and action across a range of concerns. These protocols have been examined by the ethical review board and are available on request.

### Approval

The trial has been approved by the Multi-Institutional Ethics Committee of the Anusandhan Trust, Mumbai, in sequential phases: permission for formative research in the development of the trial (February 2011), permission for slum vulnerability assessment and research on cluster gatekeepers (May 2011), permission for the baseline survey (August 2011), and permission for the intervention and evaluation component of the trial (January 2012). It has also been approved by the University College London Research Ethics Committee (reference 3546/001, January 2012).

### Communication

Since the intervention will be developed and implemented in partnership with community members, we expect communication to be regular and extensive. We will feed back descriptive research findings at meetings of community interest groups. User communities for the research findings include community members involved in resource center activities, respondents to data collection exercises, SNEHA team members, the Municipal Corporation of Greater Mumbai, the ICDS, community-based organizations, development activists and opinion formers, Indian and international academics, and students of development, health and social work. Senior government officers will be updated through regular meetings and we will arrange data sharing events for health workers. SNEHA is represented on the Family and Child Welfare Governance Council formed by the Municipal Corporation to influence program planning and implementation for reproductive and child health. Activists, opinion formers and academics will be updated on key findings through our other existing networks, which include, for example, the *Jan Swasthya Abhiyaan* and NGO federations, and the steering committee of the M Ward Transformation Project of the Tata Institute of Social Sciences. The trial findings and ancillary analyses will be published in peer-reviewed journals and presented at national and international meetings.

### Timeline

**Figure 3:**
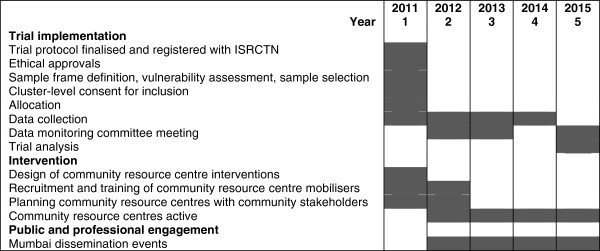
Program timeline.

The program duration is 5 years (Figure 3). Baseline data collection will run for the first 18 months, in three sequential 6-month phases. Years 2 to 4 (2012 to 2014) will be occupied with trial implementation and endline data collection. The fifth year (2015) will be used for analysis, writing up, dissemination, planning for sustainability and follow-on projects. There will be quarterly progress reviews.

## Trial status

The baseline census began in September 2011. The first phase of community resource centers opened in February 2012, and the second phase in August 2012.

## Abbreviations

DMC: Data monitoring committee; ICDS: Integrated Child Development Services; NGO: Non-government organization; NUHM: National Urban Health Mission; ODK: Open Data Kit; SD: Standard deviation; SNEHA: Society for Nutrition, Education and Health Action.

## Competing interests

The authors declare that they have no competing interests.

## Authors’ contributions

NSM, SD, UB, MR, GA, WJ, SP and DO made substantial contributions to the conception and design of the trial. DO drafted the manuscript. NSM, SD, UB, GA, MR, and SP revised the manuscript critically for intellectual content. All authors read and approved the final version.
